# Functional Complementation of Anti-Adipogenic Phytonutrients for Obesity Prevention and Management

**DOI:** 10.3390/nu14204325

**Published:** 2022-10-16

**Authors:** Yasuyo Urasaki, Thuc T. Le

**Affiliations:** College of Pharmacy, Roseman University of Health Sciences, 10530 Discovery Drive, Las Vegas, NV 89135, USA

**Keywords:** adipogenesis, functional complementation, metabolic syndrome, nutraceuticals, nutrition intervention, obesity, phytonutrients, weight management

## Abstract

Obesity is an established risk factor for metabolic disease. This study explores the functional complementation of anti-adipogenic phytonutrients for obesity prevention and management. Nine phytonutrients were selected based on their ability to affect the expression of one or more selected adipogenic biomarker proteins. The phytonutrients include berberine, luteolin, resveratrol, fisetin, quercetin, fucoidan, epigallocatechin gallate, hesperidin, and curcumin. The selected adipogenic biomarker proteins include PPARɣ, SREBP1c, FASN, PLIN1, FABP4, and β-catenin. Individually, phytonutrients had variable effects on the expression level of selected adipogenic biomarker proteins. Collectively, the functional complementation of nine phytonutrients suppressed de novo fatty acid biosynthesis via the negative regulation of PPARɣ, FASN, PLIN1, and FABP4 expression; activated glycolysis via the positive regulation of SREBP1c expression; and preserved cell–cell adhesion via the inhibition of β-catenin degradation. In primary human subcutaneous preadipocytes, the composition of nine phytonutrients had more potent and longer lasting anti-adipogenic effects compared to individual phytonutrients. In a diet-induced obesity murine model, the composition of nine phytonutrients improved glucose tolerance and reduced weight gain, liver steatosis, visceral adiposity, circulating triglycerides, low-density lipoprotein cholesterol, and inflammatory cytokines and chemokines. The functional complementation of anti-adipogenic phytonutrients provides an effective approach toward engineering novel therapeutics for the prevention and management of obesity and metabolic syndrome.

## 1. Introduction

The current obesity epidemic presents a major challenge to global health management. In the United States, the prevalence of obesity in adults was 41.9% in 2017 [1]. The estimated medical cost of obesity in the US was nearly USD 173 billion in 2019 [2]. According to the World Health Organization, 13% of adults globally, or over 600 million people, were obese in 2016. Obesity is an established risk factor for the development of type 2 diabetes mellitus and chronic inflammatory diseases, such as dyslipidemia, non-alcoholic fatty liver disease, hypertension, coronary heart disease, stroke, rheumatoid arthritis, and certain cancers [3]. Weight loss via caloric restriction and exercise is clearly the most effective strategy for the prevention of obesity and associated diseases [4]. However, achieving weight loss by lifestyle intervention can be challenging for many patients to maintain in the long term. Current FDA-approved anti-obesity drugs are moderately effective but are often associated with adverse side effects and are costly, which limit their long-term usage [5]. Alternatively, nutrition intervention provides a readily accessible means for the long-term management of obesity and associated medical conditions [6]. Phytonutrients, or natural compounds in plants and mushrooms, have anti-obesity effects by reducing appetite, modulating lipid absorption and metabolism, enhancing insulin sensitivity and thermogenesis, and inducing changes to the gut microbiota [7]. The consumption of phytonutrients is considered a safe and inexpensive approach to prevent obesity and associated conditions [7,8].

Obesity is characterized by increased adipose tissue mass via hypertrophy, which describes an increase in the size of existing fat cells or adipocytes, or hyperplasia, which describes the formation of new adipocytes from precursor cells or preadipocytes [9]. Adipogenesis is the process by which preadipocytes cells commit to the adipogenic lineage, express adipogenic proteins, accumulate intracellular lipid storage, increase cell volume, and become fully differentiated adipocytes [10,11,12,13]. Critical adipogenic proteins include peroxisome proliferator-activated receptor ɣ (PPARɣ), sterol regulatory element-binding transcription factor 1c (SREBP1c), fatty acid synthase (FASN), perilipin 1 (PLIN1), adipocyte-specific fatty acid binding protein (FABP4), and β-catenin. PPARɣ is a transcription factor that regulates fatty acid biosynthesis and glucose metabolism [14,15]. SREBP1c is a transcription factor that regulates glycolysis and sterol biosynthesis [16]. FASN is an enzyme that catalyzes de novo fatty acid synthesis [17]. PLIN1 is a lipid droplet-associated protein that regulates lipid storage [18]. FABP4 is an adipocyte-specific fatty acid binding protein that transports fatty acids [19]. β-catenin is a protein that participates in the regulation and coordination of cell–cell adhesion [20,21]. During adipogenesis, the expression of PPARɣ, SREBP1c, FASN, PLIN1, and FABP4 increases, while that of β-catenin decreases.

This study explores the functional complementation of anti-adipogenic phytonutrients for weight management. Automated and multiplexed proteomic methods are used to screen phytonutrients for their anti-adipogenicity by affecting the expression of one or more adipogenic proteins in primary human subcutaneous preadipocytes [22,23,24,25,26,27,28,29,30,31,32,33]. Specifically, phytonutrients are chosen by them meeting at least one of the following selection criteria: (1) the suppression of lipid metabolism, fatty acid transport and biosynthesis, and lipid droplet formation via the negative regulation of PPARɣ, FABP4, FASN, and PLIN1; (2) the activation of glycolysis via the positive regulation of SREBP1c expression; and (3) the preservation of cell–cell adhesion via the inhibition of β-catenin degradation. Out of more than one hundred phytonutrients screened, nine anti-adipogenic phytonutrients were selected for combination, including berberine [34], luteolin [35], resveratrol [36], fisetin [37], quercetin [38], fucoidan [39], epigallocatechin gallate [40], hesperidin [41], and curcumin [42]. Then functional complementation of these phytonutrients is designed to promote glucose metabolism while suppressing the lipid metabolism and morphological transformation of differentiating preadipocytes. A diet-induced obesity murine model is employed to assess the systemic effects of this rationally designed combination of phytonutrients.

## 2. Materials and Methods

### 2.1. Adipogenesis Assays 

Primary human subcutaneous preadipocytes were isolated from the thigh white adipose tissues of a healthy Caucasian female who was forty-three years old and had a BMI < 24.9 (cat. no. SP-F-1, Lot#L031219A, Zen-Bio, Durham, NC, USA). Fat cell differentiation was induced using a previously published protocol [43]. Briefly, preadipocytes were grown to confluence in a growth medium comprising Minimum Essential Medium α supplemented with 10% fetal bovine serum, 100 units/mL penicillin, and 100 µg/mL streptomycin. At two days post-confluence, the growth medium was aspirated off the culture dishes, and the complete differentiation medium was added (day zero, d0). The complete differentiation medium comprises DMEM/F12 with 18.5 mM glucose, HEPES (15 mM), NaHCO3 (25 mM), 100 units/mL penicillin, 100 µg/mL streptomycin, d-biotin (33 µM), pantothenate (17 µM), dexamethasone (100 nM), insulin (100 nM), rosiglitazone (1 µM), IBMX (0.5 mM), triiodothyronine (T3, 2 nM), and transferrin (10 µg/mL). On day three (d3) post-differentiation, the complete differentiation medium was replenished. On day seven (d7) post-differentiation, the complete differentiation medium was replaced with the maintenance medium. The maintenance medium comprises DMEM/F12, 100 units/mL penicillin, 100 µg/mL streptomycin, HEPES (15 mM), NaHCO3 (25 mM), d-biotin, pantothenate, insulin (10 nM), and dexamethasone (10 nM). The maintenance medium was replenished on day ten (d10) post-differentiation. The complete differentiation of preadipocytes into adipocytes was achieved on day fourteen (d14) post-differentiation.

### 2.2. Treatment Conditions

Three cell cultures were generated for the screening of each phytonutrient: a cell culture of preadipocytes on d0, a cell culture of differentiating adipocytes on d6 post-differentiation, and a cell culture of differentiating adipocytes on d6 post-differentiation in the presence of phytonutrients. Phytonutrients were added to the complete differentiation media on d0 and d3 post-differentiation. No phytonutrient was present in the maintenance media from d7 to d14. 

### 2.3. Phytonutrients 

The sources of phytonutrients and their purities are listed in Table S1 for cell culture studies and in Table S2 for animal studies. Different suppliers of phytonutrients were used for animal studies versus cell culture studies due to the food grade requirement for veterinary use. Phytonutrients from different suppliers exhibited identical effects on the expression level of six selected adipogenic biomarkers in cell cultures. The chemical structures of phytonutrients and their concentrations in cell cultures, percentages in the F1 combination, daily doses for mice, and human equivalent daily doses are listed in Table S3.

### 2.4. Preparation of Total Cell Extracts

Approximately one million cells were mixed with 60 µL of lysis buffer (cat. no. 040-764, ProteinSimple, Santa Clara, CA, USA) and incubated on ice for 10 min; then, they were sonicated four times at 5 s duration, mixed by rotation for 2 h at 4 °C, and centrifuged at 12,000 rpm in an Eppendorf 5430R microfuge for 20 min at 4 °C. The total protein concentration in the supernatant cell lysate was determined with a Bradford protein assay and adjusted to a final concentration of 0.3 µg/µL with separation gradients (cat. no. Premix G2, pH 5–8, ProteinSimple) for charge-based cIEF immunoassays or to 0.4 µg/µL with denaturing buffers (cat. no. PS-ST01EZ or PS-ST03EZ, ProteinSimple) for size-based Western immunoassays.

### 2.5. Capillary Western Immunoassays

Cell lysates were denatured at 95 °C for 5 min and then transferred to assay plates (cat. no. SM-W004 or SM-W008, ProteinSimple) preloaded with blocking reagents, wash buffer, primary and secondary antibodies, and chemiluminescent substrates. The default protocols of the Jess system (ProteinSimple) were used for sized-based protein separation and detection in capillaries. β-Actin and HSP60 were used as loading controls. Triplicate measurements for each protein and duplicate experiments were performed for each treatment condition to produce six repeated measurements per protein analyte. The expression levels of PPARγ, SREBP1c, FASN, PLIN1, and β-catenin were detected with capillary Western immunoassays.

### 2.6. Capillary Isoelectric Focusing Immunoassays

Cell lysates were loaded into 384-well assay plates (cat. no. 040-663, ProteinSimple) preloaded with primary and secondary antibodies and chemiluminescent substrates. The default protocols of the NanoPro 1000 system (ProteinSimple) were used for charge-based protein separation and detection in individual capillaries. Hsp70 was used as the loading control. Triplicate measurements for each protein and duplicate experiments were performed for each treatment condition to produce six repeated measurements per protein analyte. The expression level of FABP4 was detected with capillary isoelectric focusing immunoassays.

### 2.7. Antibodies

The primary antibodies used for this study were: PPARɣ (cat. no. CS2443, 1:50 dilution, Cell Signaling, Danvers, MA, USA), SREBP1c (cat. no. NB600-582, 1:8 dilution, Novus Biologicals, Littleton, CO, USA), FASN (cat. no. CS3189, 1:50 dilution, Cell Signaling), PLIN1 (cat. no. CS9349, 1:20 dilution, Cell Signaling), FABP4 (cat. no. AB92501, 1:50 dilution, Abcam, Cambridge, MA, USA), β-catenin (cat. no. NBPI-54467, 1:50 dilution, Novus Biologicals), β-actin (cat. no. MAB8919, 1:25 dilution, R&D Systems, Minneapolis, MN, USA), HSP60 (cat. no. F1800, 1:10 dilution, R&D Systems), and HSP70 (cat. no. 4872, 1:50 dilution, Cell Signaling). The secondary antibodies used for this study were: anti-rabbit HRP (cat. no. 040-656, 1:50 dilution & 042-206, no dilution, ProteinSimple), anti-mouse HRP (cat. no. 042-205, no dilution, ProteinSimple), anti-rabbit NIR (cat. no. 043-819, no dilution, ProteinSimple), and anti-mouse NIR (cat. no. 043-821, no dilution, ProteinSimple).

### 2.8. Diet-Induced Obesity Murine Model

C57BL/6J mice (male, approximately ten weeks old, Jackson Lab, Bar Harbor, ME, USA) were divided into three groups: a group fed with a lean diet (LD, n = 40), a group fed with a high-fat diet (HFD, n = 40), and a group fed with a high-fat diet supplemented with F1 (HFD + F1, n = 40). The lean diet (cat. no. TD7001, Teklad Diets, Madison, WI, USA) comprised proteins (25.2% by weight), carbohydrates (39.5% by weight), fats (4.4% by weight), and others (30.9% by weight, ash, fibers, others). The lean diet has 3 kcal/g, with 34% of kcal from proteins, 53% of kcal from carbohydrates, and 13% of kcal from fats. The high-fat diet (cat. no. TD88137, Teklad Diets) comprised proteins (17.3% by weight), carbohydrates (48.5% by weight), fats (21.2% by weight), and others (13% by weight, ash, fibers, others). The high-fat diet has 4.5 g/kcal, with 15.2% of kcal from proteins, 42.7% of kcal from carbohydrates, and 42% of kcal from fats. F1 was supplemented at 0.1% by weight, leading to an approximate daily dose of 200 mg/kg for mice, or approximately 16 mg/kg of a human equivalent dose. The mice groups were placed on their respective diets for seventeen weeks. Glucose tolerance tests were performed at week sixteen. Terminal tissue and blood samples collection was performed at week seventeen. The histology of the liver and visceral adipose tissues was performed by IHC WORLD (Woodstock, MD, USA). Blood triglycerides, cholesterol, HDL, and LDL were measured by IDEXX Analytics (West Sacramento, CA, USA). The Proteome Profiler Mouse Cytokine Array Kits (cat. no. ARY006, R&D Systems, Minneapolis, MN, USA) were used to measure inflammatory cytokines in collected blood samples. All animal studies were performed in accordance with the Public Health Service Policy on the Humane Care and Use of Laboratory Animals and with the approval of the Animal Care and Use Committee at Roseman University of Health Sciences.

### 2.9. Glucose Tolerance Test

The mice were housed in cages that did not have bedding that could serve as alternative food sources. The mice were fasted overnight for 16 h, with access to water. The morning following fasting, the mice were anesthetized with 1.5–4% isoflurane at a flow rate of 1 L/min. The tail tips were clipped with a pair of scissors and wiped off with alcohol gauzes. Blood was collected at 0 min for measurement with a glucometer. Next, the mice were injected with 2 g dextrose per kg body mass using 27G needles. The volume of injected dextrose was calculated using the following formula: 50% dextrose (µL) = 4 × body weight (g). Blood was collected at 30, 60, 90, and 120 min after injection for the determination of glucose levels. Between each of these time points, the mice were returned to their cages and monitored continuously. At the end of the glucose tolerance tests, the mice were returned to their previous housing and feeding conditions. 

### 2.10. Confocal Laser Scanning Microscopy

Primary human subcutaneous preadipocytes were cultured on uncoated 55 mm glass-bottom dishes for confocal fluorescence imaging (cat. no. p50G-1.5-30-F, MatTek, Ashland, MA, USA). The cells were fixed with 5% formaldehyde and stained for the nucleus with Hoechst 33,258 (cat. no. H3569, Invitrogen, Eugene, OR, USA), F-actin with Alexa Fluor 488 phalloidin (cat. no. A12379, Invitrogen), tubulin with paclitaxel Bodipy 564/570 conjugate (cat. no. P7501, Invitrogen), and neutral lipids with HCS LipidTOX™ Deep Red (cat. no. H34477, Invitrogen). A Nikon A1R inverted confocal microscope with a 60X oil-immersion objective and a numerical aperture of 1.49 was used for imaging. Solid-state lasers at 405, 488, 561, and 638 nm were used for excitation, and fluorescence emissions were collected using 425–475, 500–550, 570–620, and 662–737 nm filters.

### 2.11. Statistical Analysis

Statistical significance was calculated using Excel software (Microsoft, Redmond, WA, USA) and Student’s paired t-test with two-tailed distribution and a threshold at *p*-value ≤ 0.01 versus the control.

## 3. Results

### 3.1. Suppression of PPARɣ Expression

**Figure 1:**
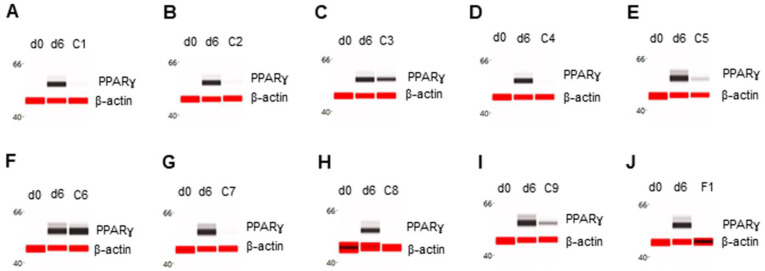
Suppression of PPARγ expression in differentiating preadipocytes. Expression level of PPARγ in preadipocytes on d0, d6 post-differentiation (d6), and d6 post-differentiation in the presence of (**A**) berberine (C1), (**B**) luteolin (C2), (**C**) resveratrol (C3), (**D**) fisetin (C4), (**E**) quercetin (C5), (**F**) fucoidan (C6), (**G**) EGCG (C7), (**H**) hesperidin (C8), (**I**) curcumin (C9), or (**J**) F1 composition. β-actin served as a loading control. The protein expression level was measured using multiplexed capillary Western immunoassays. Molecular markers in kilodalton (kDa) are displayed on the left of the capillary Western immunoassay data.

The effect of phytonutrients on the expression of adipogenic marker proteins was examined in primary human subcutaneous preadipocytes undergoing fat cell differentiation. Briefly, post-confluent preadipocytes were induced to undergo adipogenesis via the addition of complete differentiation media for six days in the absence or presence of phytonutrients. On d6, total cell extracts were collected, and the expression levels of six adipogenic protein biomarkers were analyzed using capillary Western immunoassays or capillary isoelectric focusing immunoassays. The expression level of the PPARɣ transcription factor was undetectable in preadipocytes on d0 and highly expressed in differentiating preadipocytes on d6. Individually, phytonutrients exhibited differential effects on the expression of adipogenic protein biomarkers. The phytonutrients berberine, luteolin, fisetin, quercetin, EGCG, hesperidin, and curcumin suppressed PPARɣ expression in differentiating preadipocytes (Figure 1A,B,D,E,G–J). The phytonutrients resveratrol and fucoidan had no effect on PPARɣ expression in differentiating preadipocytes (Figure 1C,F). Collectively, F1 composition suppressed PPARɣ expression in differentiating preadipocytes (Figure 1J). 

### 3.2. Enhancement of SREBP1c Expression

**Figure 2:**
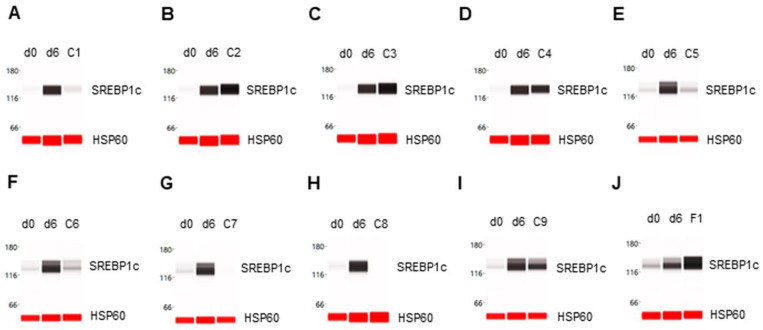
Enhancement of SREBP1c expression in differentiating preadipocytes. Expression level of SREBP1c in preadipocytes on d0, d6 post-differentiation, and d6 post-differentiation in the presence of (**A**) berberine (C1), (**B**) luteolin (C2), (**C**) resveratrol (C3), (**D**) fisetin (C4), (**E**) quercetin (C5), (**F**) fucoidan (C6), (**G**) EGCG (C7), (**H**) hesperidin (C8), (**I**) curcumin (C9), or (**J**) F1 composition. β-actin served as a loading control. The protein expression level was measured using multiplexed capillary Western immunoassays. Molecular markers in kilodalton (kDa) are displayed on the left of the capillary Western immunoassay data.

The expression level of the SREBP1c transcription factor was undetectable in preadipocytes on d0 and highly expressed in differentiating preadipocytes on d6. The phytonutrients luteolin and resveratrol enhanced SREBP1c expression in differentiating preadipocytes (Figure 2B,C). The phytonutrients berberine, quercetin, fucoidan, EGCG, and hesperidin suppressed SREBP1c expression in differentiating preadipocytes (Figure 2A,E,F,G,H). The phytonutrients fisetin and curcumin had no significant effect on SREBP1c expression (Figure 2C,I). Collectively, F1 composition substantially enhanced SREBP1c expression in differentiating preadipocytes (Figure 2J). 

### 3.3. Suppression of FASN Expression

**Figure 3:**
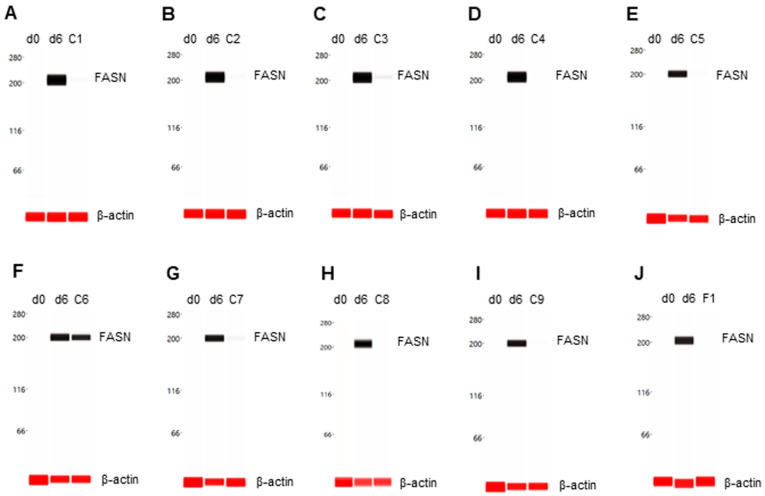
Suppression of FASN expression in differentiating preadipocytes. Expression level of FASN in preadipocytes on d0, d6 post-differentiation, or d6 post-differentiation in the presence of (**A**) berberine (C1), (**B**) luteolin (C2), (**C**) resveratrol (C3), (**D**) fisetin (C4), (**E**) quercetin (C5), (**F**) fucoidan (C6), (**G**) EGCG (C7), (**H**) hesperidin (C8), (**I**) curcumin (C9), or (**J**) F1 composition. β-actin served as a loading control. The protein expression level was measured using multiplexed capillary Western immunoassays. Molecular markers in kilodalton (kDa) are displayed on the left of the capillary Western immunoassay data.

The expression level of FASN was undetectable in preadipocytes on d0 and highly expressed in differentiating preadipocytes on d6. Eight phytonutrients, including berberine, luteolin, resveratrol, fisetin, quercetin, EGCG, hesperidin, and curcumin, suppressed FASN expression in differentiating preadipocytes (Figure 3A–E,G–I). The phytonutrient fucoidan had no effect on FASN expression in differentiating preadipocytes (Figure 3F). Collectively, F1 composition suppressed FASN expression in differentiating preadipocytes (Figure 3J). 

### 3.4. Suppression of PLIN1 Expression

**Figure 4:**
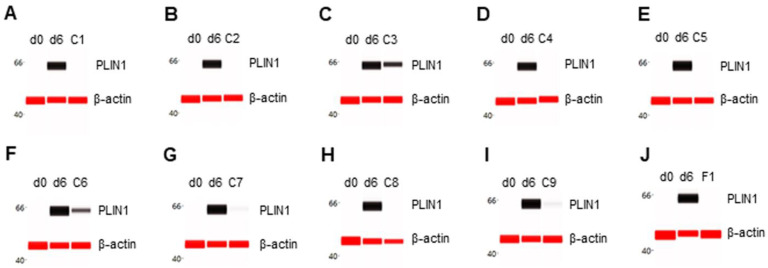
Suppression of PLIN1 expression in differentiating preadipocytes. Expression level of PLIN1 in preadipocytes on d0, d6 post-differentiation, or d6 post-differentiation in the presence of (**A**) berberine (C1), (**B**) luteolin (C2), (**C**) resveratrol (C3), (**D**) fisetin (C4), (**E**) quercetin (C5), (**F**) fucoidan (C6), (**G**) EGCG (C7), (**H**) hesperidin (C8), (**I**) curcumin (C9), or (**J**) F1 composition. β-actin served as a loading control. The protein expression level was measured using multiplexed capillary Western immunoassays. Molecular markers in kilodalton (kDa) are displayed on the left of the capillary Western immunoassay data.

The expression level of PLIN1 was undetectable in preadipocytes on d0 and highly expressed in differentiating preadipocytes on d6. All selected phytonutrients suppressed PLIN1 expression in differentiating preadipocytes (Figure 4A–I). Consistently, F1 composition also suppressed PLIN1 expression in differentiating preadipocytes (Figure 4J). 

### 3.5. Suppression of FABP4 Expression

**Figure 5:**
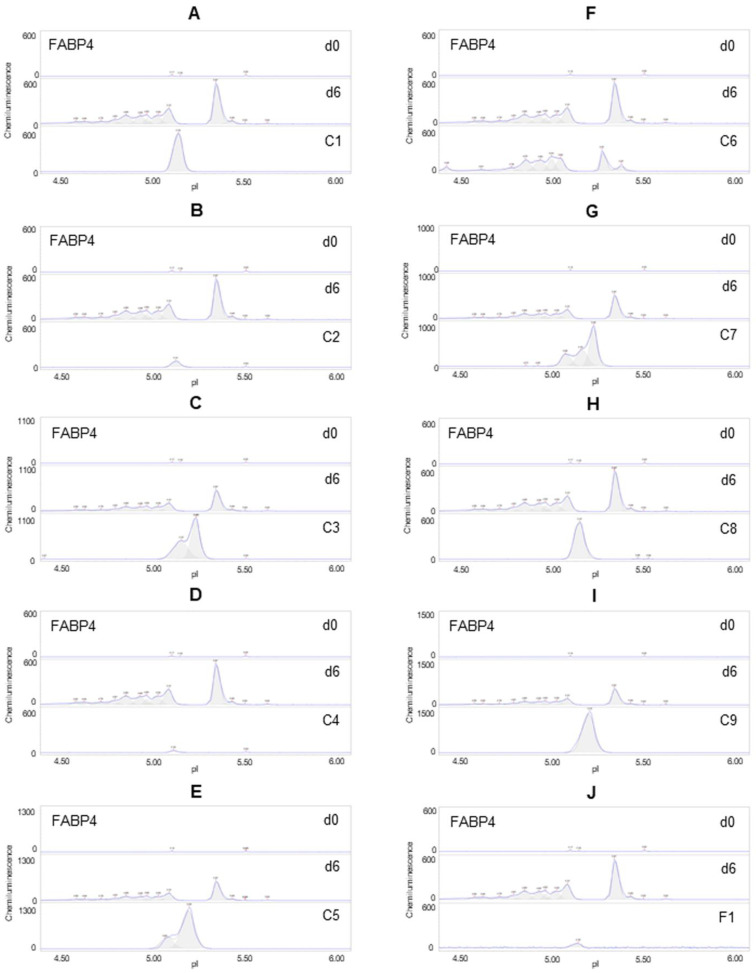
Suppression of FAB4 expression in differentiating preadipocytes. Expression level of FABP4 in preadipocytes on d0, d6 post-differentiation (d6), or d6 post-differentiation in the presence of (**A**) berberine (C1), (**B**) luteolin (C2), (**C**) resveratrol (C3), (**D**) fisetin (C4), (**E**) quercetin (C5), (**F**) fucoidan (C6), (**G**) EGCG (C7), (**H**) hesperidin (C8), (**I**) curcumin (C9), or (**J**) F1 composition. β-actin served as a loading control. The FABP4 protein post-translational modification profile was measured using capillary isoelectric focusing immunoassays.

The expression level of FABP4 was undetectable in preadipocytes on d0 and highly expressed in differentiating preadipocytes on d6. Six phytonutrients, including berberine, resveratrol, quercetin, EGCG, hesperidin, and curcumin, did not suppress the expression of FABP4 but reduced its distribution as a function of isoelectric points (Figure 5A,C,E,G–I). The phytonutrients luteolin and fisetin suppressed FABP4 expression in differentiating preadipocytes (Figure 5B,D). The phytonutrient fucoidan had no effect on FABP4 expression or pI distribution (Figure 5F). Collectively, F1 composition suppressed FFABP4 expression in differentiating preadipocytes (Figure 5J). 

### 3.6. Inhibition of β-Catenin Degradation

**Figure 6:**
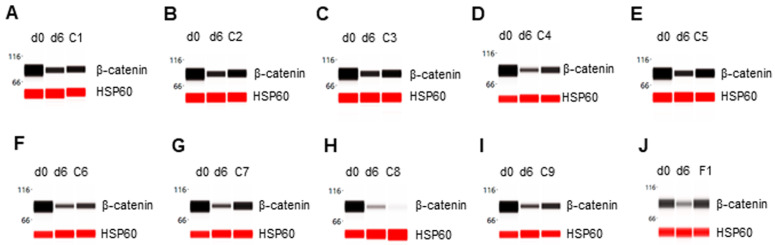
Inhibition of β-catenin degradation in differentiating preadipocytes. Expression level of β-catenin in preadipocytes on d0, d6 post-differentiation, or d6 post-differentiation in the presence of (**A**) berberine (C1), (**B**) luteolin (C2), (**C**) resveratrol (C3), (**D**) fisetin (C4), (**E**) quercetin (C5), (**F**) fucoidan (C6), (**G**) EGCG (C7), (**H**) hesperidin (C8), (**I**) curcumin (C9), or (**J**) F1 composition. β-actin served as a loading control. The protein expression level was measured using multiplexed capillary Western immunoassays. Molecular markers in kilodalton (kDa) are displayed on the left of the capillary Western immunoassay data.

The expression level of β-catenin was high in preadipocytes on d0 and strongly repressed in differentiating preadipocytes on d6. Three phytonutrients, including berberine, fucoidan, and hesperidin, did not interfere with β-catenin degradation in differentiating preadipocytes (Figure 6A,F,H). Six remaining phytonutrients, including luteolin, resveratrol, fisetin, quercetin, EGCG, and curcumin, moderately inhibited β-catenin degradation in differentiating preadipocytes (Figure 6B–E,G,I). Collectively, the F1 composition of phytonutrients suppressed FABP4 expression in differentiating preadipocytes (Figure 5J). Table 1 summarizes the expression level of adipogenic biomarker proteins as functions of individual phytonutrients or F1 composition. 

### 3.7. Potent and Lasting Inhibition of Adipogenesis by F1 Composition 

**Figure 7:**
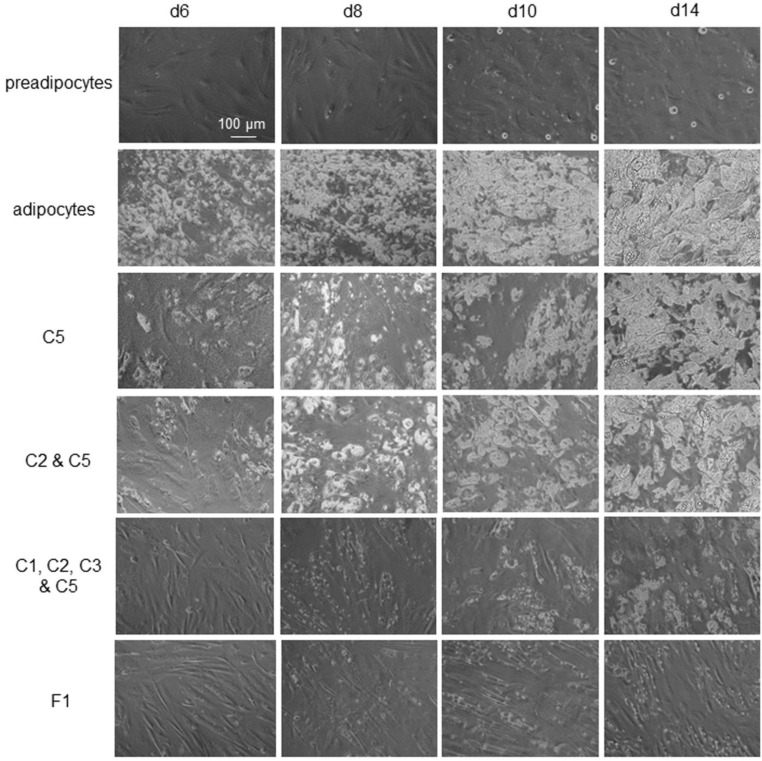
Potent and lasting inhibition of adipogenesis by F1 composition. First row: Undifferentiated preadipocytes in growth media. Second row: Differentiating preadipocytes or adipocytes. Third row: Differentiating preadipocytes in complete differentiation media supplemented with quercetin (C5). Fourth row: Differentiating preadipocytes in complete differentiation media supplemented with luteolin (C2) and quercetin (C5). Fifth row: Differentiating preadipocytes in complete differentiation media supplemented with berberine (C1), luteolin (C2), resveratrol (C3), and quercetin (C5). Sixth row: Differentiating preadipocytes in complete differentiation media supplemented with the F1 composition of phytonutrients. First column: d6 post-differentiation; second column: d8 post-differentiation; third column: d10 post-differentiation; fourth column: d14 post-differentiation. Cytoplasmic lipid droplets appeared as white particles under phase-contrast microscopy.

All selected phytonutrients in this study were anti-adipogenic phytonutrients. Their anti-adipogenicity was most prominent when they were present in differentiating media from d0 to d6 (Figure 7, first column). Following their removal from culturing media on d7, the anti-adipogenic effects waned rapidly, and intracellular lipid droplet accumulation accelerated in differentiating adipocytes. On d14 post-differentiation, the intracellular lipid droplet content was indistinguishable between differentiating adipocytes treated with individual phytonutrients and untreated control differentiating adipocytes (Figure 7, second and third rows, last column). Combinations of phytonutrients improved the potency and durability of anti-adipogenic effects. Specifically, combinations of four or more phytonutrients exhibited observable anti-adipogenic effects on d14 post-differentiation or seven days after the removal of phytonutrients (Figure 7, fifth row, last column). The most potent and lasting anti-adipogenic effect was observed with the F1 composition (Figure 7, last row). F1 composition suppressed cytoplasmic lipid accumulation by 100% at d6 post-differentiation and by more than 95% on d14 post-differentiation (Figure S1). Most interestingly, differentiating preadipocytes maintained spindle cell morphology through two weeks of differentiation.

### 3.8. Inhibition of Intracellular Lipid Droplet Accumulation by F1 Composition 

**Figure 8:**
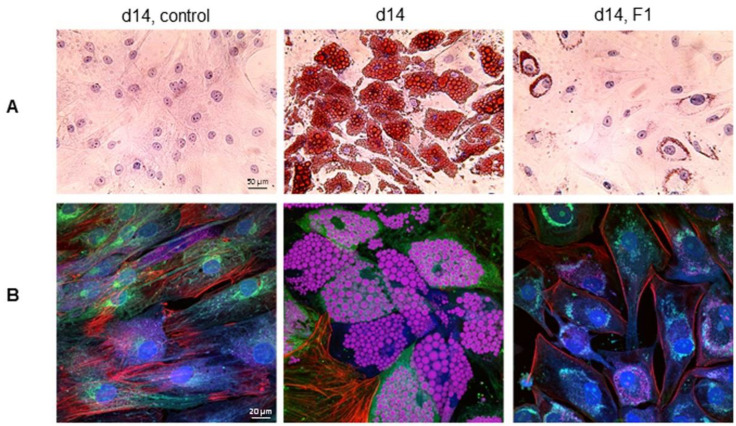
Inhibition of intracellular lipid droplet accumulation by the F1 composition of phytonutrients. (**A**) Brightfield images of hematoxylin-, eosin-, and Oil Red O-stained control undifferentiated preadipocytes (left panel), control differentiating adipocytes on d14 post-differentiation (middle panel), and differentiating adipocytes treated with F1 composition on d14 post-differentiation (right panel). (**B**) Multi-color confocal fluorescence images of control undifferentiated preadipocytes (left panel), control differentiating adipocytes on d14 post-differentiation (middle panel), and differentiating adipocytes treated with F1 composition on d14 post-differentiation (right panel). Cells were stained for nucleus (blue), actin (green), tubulin (red), and neutral lipids (pink).

The high-resolution imaging of intracellular lipid droplets by independent methods further confirmed the anti-adipogenic effects of the F1 composition of phytonutrients. Following hematoxylin, eosin, and Oil Red O staining, brightfield microscopy revealed the minor Oil Red O staining of intracellular lipid droplets on d14 in differentiating adipocytes treated with the F1 composition of phytonutrients versus untreated control (Figure 8A). Furthermore, multi-color confocal fluorescence imaging confirmed the lack of intracellular lipid droplets on d14 post-differentiation in differentiating adipocytes treated with the F1 composition of phytonutrients versus untreated control (Figure 8B). 

### 3.9. Dietary Supplement with F1 Composition Reduces Weight Gain

**Figure 9:**
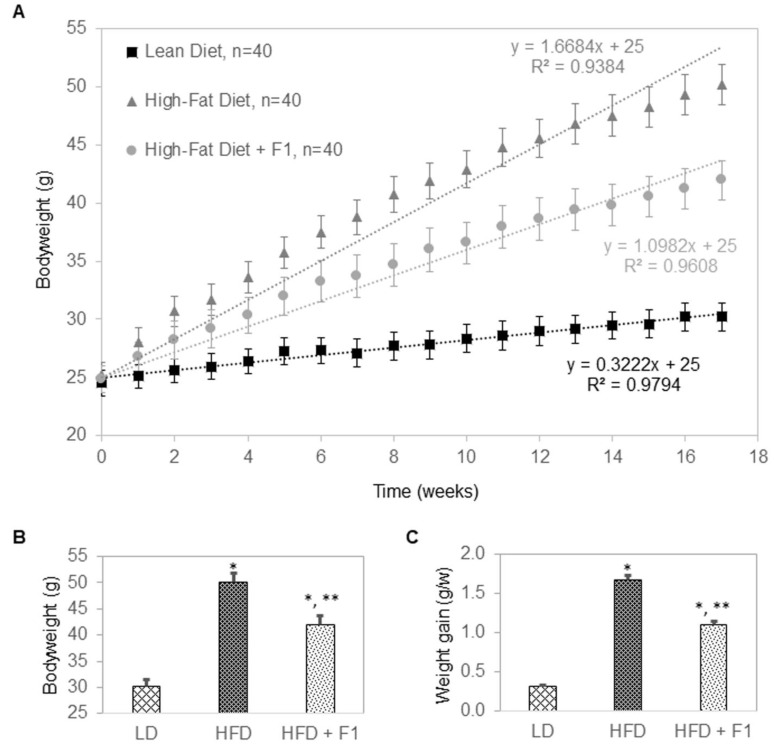
F1 composition reduces weight gain in diet-induced obesity mice. (**A**) Average bodyweight as a function of time of LD, HFD, and HFD + F1 animal groups. (**B**) Average bodyweight at week seventeen as a function of LD, HFD, and HFD + F1 animal groups. (**C**) Average rate of weight gain in grams per week (g/w) as a function of LD, HFD, and HFD + F1 animal groups. Error bars are standard deviations of forty mice per animal group. Single and double asterisks indicate a statistical significance of *p*-value ≤ 0.01 versus LD and HFD animal groups, respectively.

The anti-adipogenic effects of F1 composition were further evaluated in a diet-induced obesity murine model (Figure 9A–C). Mice were divided into three groups: a lean diet group (LD, n = 40), a high-fat diet group (HFD, n = 40), and a high-fat diet supplemented with F1 composition group (HFD + F1, n = 40). All mice were placed on their respective diets for seventeen weeks. At the beginning of the experiments, all mice had bodyweights of approximately 25 g (Figure 9A). LD mice gained weight at a steady rate of 0.3 g per week and reached an average of 30 g in bodyweight at the end of the study (Figure 9B,C). HFD mice gained weight rapidly at a rate of 1.7 g per week and reached an average of 50 g in bodyweight at the end of the study. Interestingly, HFD + F1 mice gained weight a rate of 1.1 g per week and reached an average of 42 g in bodyweight at the end of the study. On average, F1 composition reduced the weight gain of diet-induced obesity mice by approximately 45%. 

### 3.10. Dietary Supplement with F1 Composition Improves Glucose Tolerance

**Figure 10:**
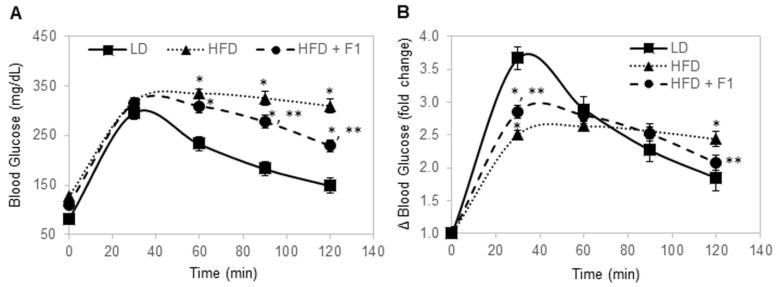
F1 composition improves glucose tolerance in diet-induced obesity mice. (**A**) Blood glucose level as a function of time post-injection. (**B**) Fold change in blood glucose level as a function of time post-injection. Error bars are the standard deviations of forty mice per animal group. Single and double asterisks indicate a statistical significance of a *p*-value ≤ 0.01 versus the LD and HFD animal groups, respectively.

Glucose tolerance tests were performed for all mice after 16 h of overnight fasting (Figure 10A). The fasting blood glucose levels were 81 mg/dL, 127 mg/dL, and 110 mg/dL for LD, HFD, and HFD + F1 mice, respectively. Following the intraperitoneal injection of 20% glucose, blood samples were collected via the tail veins and measured for blood glucose levels. The LD mice exhibited the highly dynamic regulation of the blood glucose level (Figure 10B). In the LD mice, blood glucose increased by nearly four-fold, peaked at around 30 min post-injection, and steadily declined to slightly less than two-fold higher than the baseline blood glucose level at 120 min post-injection. In contrast, the HFD mice were unable to dynamically regulate the blood glucose level. In HFD mice, blood glucose increased by two-and-a-half-fold at 30 min post-injection and stayed elevated at around two-and-a-half-fold higher than the baseline blood glucose level until 120 min post-injection. Interestingly, the HFD + F1 mice had better dynamic control of the blood glucose level compared to the HFD mice. Following glucose injection, blood glucose increased by three-fold at 30 min post-injection and steadily declined to around two-fold higher than the baseline blood glucose level at 120 min post-injection. Dietary supplement with F1 composition improved glucose tolerance in diet-induced obesity mice.

### 3.11. Dietary Supplement with F1 Composition Reduces Blood Lipids 

**Figure 11:**
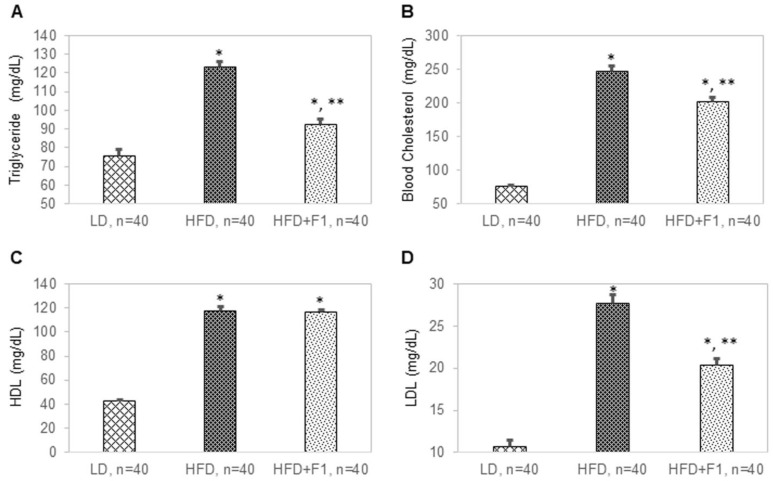
F1 composition reduces blood lipids. Blood (**A**) triglyceride, (**B**) cholesterol, (**C**) high-density lipoprotein (HDL), and (**D**) low-density lipoprotein (LDL) levels as functions of LD, HFD, and HFD + F1 animal groups. Error bars are the standard deviations of forty mice per animal group. Single and double asterisks indicate a statistical significance of a *p*-value ≤ 0.01 versus the LD and HFD animal groups, respectively.

Blood and tissue samples were terminally collected from all mice at the end of this study. The average blood triglyceride levels were 76 mg/dL, 123 mg/dL, and 93 mg/dL for the LD, HFD, and HFD + F1 mice, respectively (Figure 11A). The average total cholesterol levels were 76 mg/dL, 246 mg/dL, and 202 mg/dL for the LD, HFD, and HFD + F1 mice, respectively (Figure 11B). The average blood high-density lipoprotein (HDL) cholesterol levels were 43 mg/dL, 117 mg/dL, and 117 mg/dL for the LD, HFD, and HFD + F1 mice, respectively (Figure 11C). The average blood low-density lipoprotein (LDL) cholesterol levels were 11 mg/dL, 28 mg/dL, and 20 mg/dL for the LD, HFD, and HFD + F1 mice, respectively (Figure 11D). On average, dietary supplement with F1 composition reduced blood triglyceride and LDL cholesterol levels in diet-induced obesity mice.

### 3.12. Dietary Supplement with F1 Composition Reduces Liver Steatosis and Visceral Adiposity

**Figure 12:**
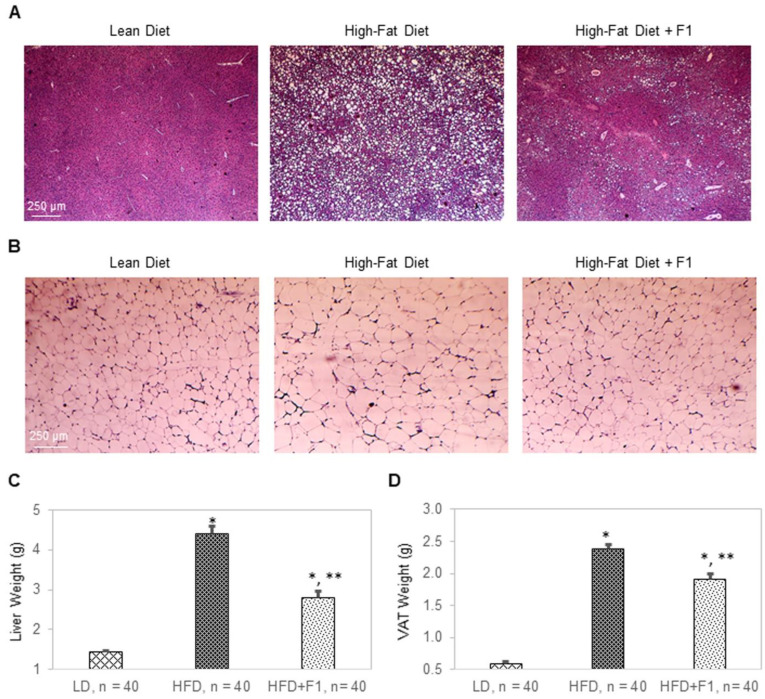
F1 composition reduces liver steatosis and visceral adiposity. H&E histology of (**A**) liver tissues and (**B**) visceral adipose tissues collected from LD, HFD, and HFD + F1 mice. Images were acquired with brightfield microscopy. Lipid droplets are indicated as white dots. Average weight of (**C**) liver tissues and (**D**) visceral adipose tissues (VAT) as functions of animal groups on specified diets. Error bars are the standard deviations of forty mice per animal group. Single and double asterisks indicate a statistical significance of a *p*-value ≤ 0.01 versus LD and HFD animal groups, respectively.

Terminally collected liver and visceral adipose tissues were used for hematoxylin and eosin (H&E) histology (Figure 12A,B). H&E histology revealed a complete absence of any lipid droplet accumulation in LD liver tissues, severe lipid droplet accumulation in HFD liver tissues, and substantially reduced levels of lipid droplet accumulation in HFD + F1 liver tissues compared to those of HFD liver tissues (Figure 12A). On average, the liver weights were approximately 1.5 g, 4.4 g, and 2.8 g for the LD, HFD, and HFD + F1 animal groups, respectively (Figure 12C). Furthermore, H&E histology revealed an average diameter of lipid droplets of visceral adipocytes that was two times higher for the HFD animal group versus the LD animal group (Figure 12B and Figure S2). On the other hand, the average diameter of lipid droplets of visceral adipocytes was one-and-a-half times higher for the HFD + F1 animal group versus the LD animal group. Consistently, the average visceral adipose tissue weights were 0.58 g, 2.4 g, and 1.9 g for the LD, HFD, and HFD + F1 animal groups, respectively (Figure 12D). H&E histology revealed that dietary supplement with F1 reduced liver steatosis and visceral adiposity in diet-induced obesity mice.

### 3.13. Dietary Supplement with F1 Composition Reduces Systemic Inflammation

**Figure 13:**
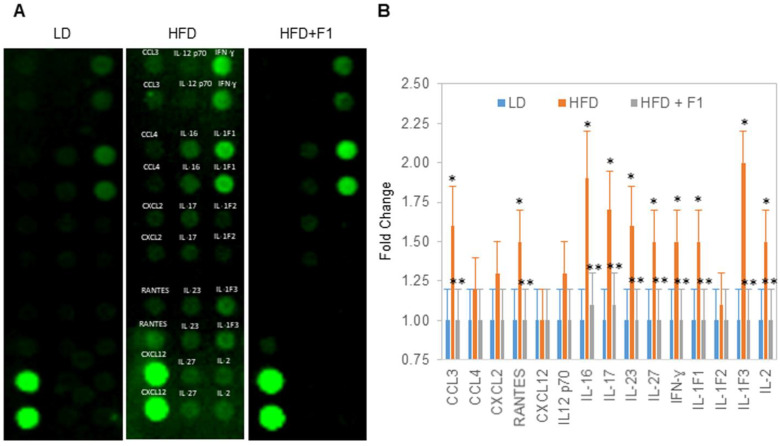
F1 composition reduces systemic inflammation. (**A**) Detection of cytokines and chemokines in the serum of LD (leftmost panel), HFD (middle panel), and HFD + F1 (right panel) animal groups. (**B**) Fold change in the relative abundance of serum cytokines and chemokines in HFD and HFD + F1 animal groups versus LD animal group. Error bars are the standard deviations of forty mice per animal group. Single and double asterisks (*) indicate a statistical significance of a *p*-value ≤ 0.01 versus LD and HFD animal groups, respectively.

Terminally collected blood samples were used to measure inflammatory chemokines and cytokines (Figure 13A). Substantial increases in the serum chemokines CCL3 and RANTES and the cytokines IL-IF1, IL-1F3, IL-2, IL-16, IL-17, IL-23, IL-27, and IFN-ɣ were detected in the HFD animal group versus the LD animal group (Figure 13B). Interestingly, the same abundances of serum cytokines and chemokines were detected for the HFD + F1 and LD animal groups. 

## 4. Discussion

Fat cell differentiation is a complex and highly regulated process [11]. The activation of a master regulator transcription factor, PPARɣ, is required to turn on the transcription of important lipid metabolism genes [15]. The insulin-induced expression of SREBP1c transcription factor is necessary to activate the transcription of genes controlling glycolysis, which provides the building blocks for de novo fatty acid biosynthesis [16,44]. Expressions of FASN, FABP4, and PLIN1 proteins are vital to adipogenesis. FASN is a mutimeric protein that catalyzes de novo fatty acid biosynthesis [45]. FABP4 is an adipocyte-specific transport protein that shuttles long-chain fatty acid to and from the surfaces of lipid droplets [46]. PLIN1 provides scaffolding structures that stabilize lipid droplets [47]. In addition, the morphological transformation of preadipocytes from a spindle to round shape and cell volume expansion are necessary to accommodate the growing lipid droplets [48]. These processes require the reduction of the cell–cell adhesion and degradation of β-catenin protein [20,21]. 

Herein, nine anti-adipogenic phytonutrients were selected based on their effects on the expression of six adipogenic biomarker proteins: PPARɣ, SREBP1c, FASN, FABP4, PLIN1, and β-catenin. The phytonutrients include berberine, luteolin, resveratrol, fisetin, quercetin, fucoidan, EGCG, hesperidin, and curcumin. The anti-adipogenicity of these phytonutrients had been reported in the literature and was further confirmed by this study [34,35,36,37,38,39,40,41,42]. Individually, these phytonutrients were highly capable of suppressing adipogenesis while present in the differentiation media up to d6 post-differentiation. However, following their removal from the culturing media on d7 post-differentiation, intracellular lipid droplet accumulation quickly resumed. By d14 post-differentiation, the intracellular lipid droplet contents of maturing adipocytes were indistinguishable between cell cultures treated with individual phytonutrients and those without treatment. 

Combinations of phytonutrients improved both the potency and duration of anti-adipogenicity. Notably, the F1 composition of all nine phytonutrients exhibited highly potent and lasting anti-adipogenicity. In primary human subcutaneous preadipocytes, F1 composition suppressed lipid metabolism, fatty acid transport and biosynthesis, and lipid droplet formation via the negative regulation of PPARɣ, FABP4, FASN, and PLIN1. F1 composition activated glycolysis via the positive regulation of SREBP1c expression. In addition, F1 composition preserved cell–cell adhesion via the inhibition of β-catenin degradation. Most interestingly, F1 composition continued to inhibit intracellular lipid droplet accumulation up to seven days following its removal from the culturing media. The functional complementarity of phytonutrients in F1 composition plausibly exerted multi-targeted effects, suppressed the expression of multiple adipogenic proteins, and delayed the resumption of cytoplasmic lipid droplet accumulation. 

In a diet-induced obesity murine model, dietary supplement with F1 composition improved glucose tolerance and reduced weight gain, liver steatosis, visceral adiposity, circulating triglycerides, LDL cholesterol, and inflammatory cytokines and chemokines. Obesity is associated with visceral adiposity, liver steatosis, hyperlipidemia, hypercholesterolemia, systemic low-grade chronic inflammation, and increased risks for the development of metabolic disease [49,50]. Dietary supplement with F1 composition has a potential for the management of obesity and associated metabolic conditions. The initial data presented herein support future clinical assessments to validate the therapeutic potential of nutrition intervention using F1 composition for obesity and metabolic disease management. 

The bioavailability assessment of F1 composition will be technically challenging due to the presence of many phytonutrients. Fortunately, high-throughput in vitro absorption, distribution, metabolism, and excretion (HT-ADME) screening and in vivo drug metabolism pharmacokinetics (DMPK) have become an essential part of the drug discovery process of synthetic molecules over the last two decades [51,52]. The future application of HT-ADME screening and DMPK for phytonutrients could facilitate their translation into the realm of food–drug nutraceuticals [53]. 

Targeting adipogenesis is a rational therapeutic approach for obesity prevention and management [5,54]. Numerous studies have reported anti-adipogenic properties of phytonutrients and classified them based on affected pathways [54,55,56,57,58]. Similarly, this study deployed multiplexed proteomic methods to identify the functional complementarity of phytonutrients based on their effects on specific adipogenic protein biomarkers. A rationally designed combination of phytonutrients achieved precise intended effects and increased anti-adipogenic potency and duration at low concentrations of individual phytonutrients. Future molecularly guided combinations of anti-adipogenic phytonutrients have the potential to further improve therapeutic precision and efficacy.

## 5. Conclusions

The functional complementation of anti-adipogenic phytonutrients increased the potency and duration of anti-adipogenicity in primary human subcutaneous preadipocytes. Dietary supplement with the F1 composition of nine anti-adipogenic phytonutrients—berberine, luteolin, resveratrol, fisetin, quercetin, fucoidan, EGCG, hesperidin, and curcumin—was highly effective at reducing weight gain, liver steatosis, visceral adiposity, circulating triglycerides, LDL cholesterol, and inflammatory cytokines and chemokines in a diet-induced obesity murine model. The functional complementation of anti-adipogenic phytonutrients provides an effective approach toward engineering novel therapeutics for the prevention and management of obesity and metabolic syndrome.

## 6. Patents

The authors are co-inventors of a U.S. patent application regarding a composition of phytonutrients for the prevention of obesity (17/741,157). 

## Figures and Tables

**Table 1:** Expression level of adipogenic biomarker proteins as functions of phytonutrients.

Biomarkers	d0	d6	C1	C2	C3	C4	C5	C6	C7	C8	C9	F1
PPARɣ	_	+	_	_	+	_	_	+	_	_	_	_
SREBP1c	_	+	_	+	+	+	_	_	_	_	+	+
FASN	_	+	_	_	_	_	_	+	_	_	_	_
PLIN1	_	+	_	_	_	_	_	_	_	_	_	_
FABP4	_	+	+	_	+	_	+	+	+	+	+	_
β-catenin	+	_	_	+	+	+	+	_	+	_	+	+

Table legend: d0: pre-differentiation; d6: post-differentiation; d6 post-differentiation in the presence of C1: berberine; C2: luteolin; C3: resveratrol; C4: fisetin; C5: quercetin; C6: fucoidan; C7: EGCG; C8: hesperidin; C9: curcumin; or F1: composition of C1–C9; _: low expression level; +: high expression level.

## Data Availability

All data presented in this study are available from the corresponding author upon reasonable request.

## Supplementary Materials

The following supporting information can be downloaded at: https://www.mdpi.com/article/10.3390/nu14204325/s1, Figure S1: Anti-adipogenic effects of individual and combinations of phytonutrients on d14; Figure S2: Average adipocyte diameter as a function of diet groups; Table S1: Phytonutrients for cell cultures; Table S2: Phytonutrients for dietary supplement; Table S3: Chemical structures, concentrations, and daily doses of phytonutrients.
