# Supplementary material for: Functional Complementation of Anti-Adipogenic Phytonutrients for Obesity Prevention and Management
Source: Nutrients. 2022 Oct 16;14(20):4325. doi: 10.3390/nu14204325 (PMC9609749; doi:10.3390/nu14204325)
Supplement: Supplementary file 1 [file nutrients-14-04325-s001.zip › nutrients-1961585-supplementary.pdf]

Supplementary Materials

Functional Complementation of Anti-Adipogenic Phytonutrients for Obesity Prevention and Management

Yasuyo Urasaki and Thuc T. Le *

College of Pharmacy, Roseman University of Health Sciences, 10530 Discovery Drive, Las Vegas, NV 89135, USA

*Correspondence: Thuc T. Le, tle5@roseman.edu

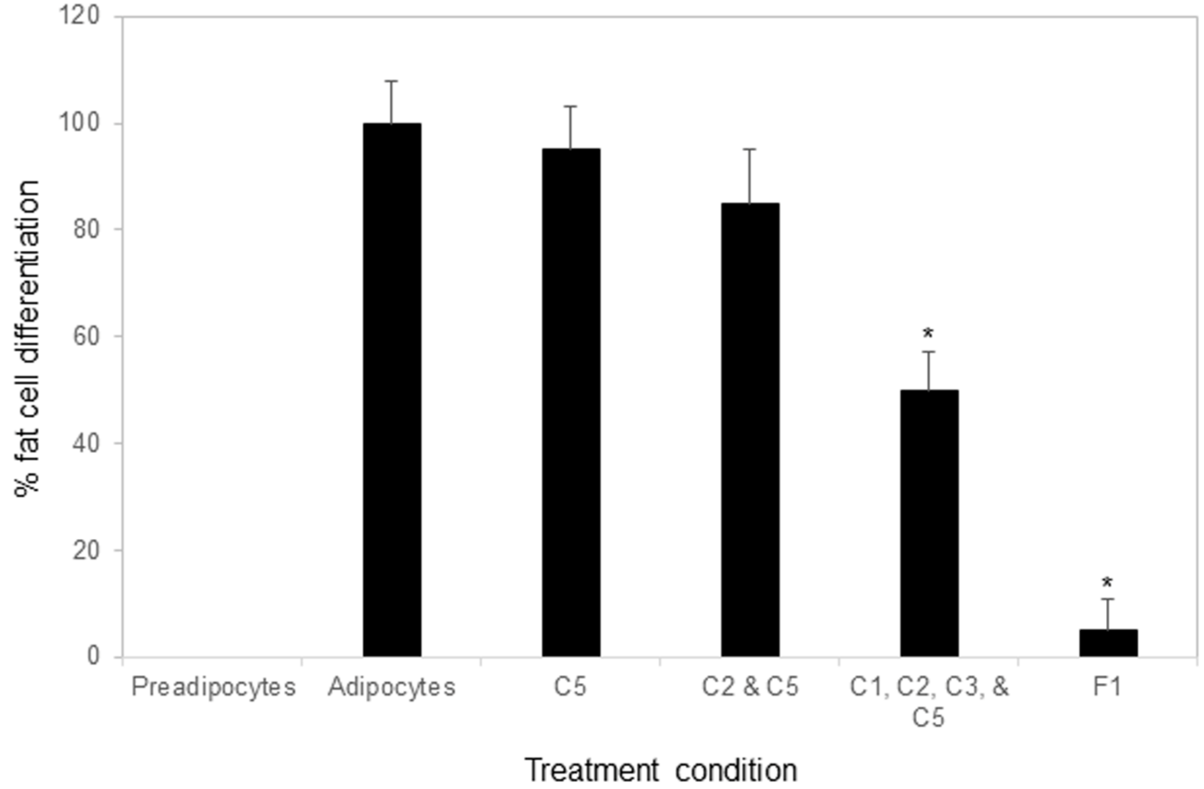

Figure S1. Anti-adipogenic effects of individual and combinations of phytonutrients on d14. H&E- and ORO-stained images were visually examined and manually counted for the number of differentiated cells as a function of total cells, which included differentiated and undifferentiated cells, in each 600 x 450 μm field of view. Hematoxylin-stained nuclei provided the number of total cells and ORO-stained cells provided the number of differentiated cells. At least ten field-of-views were used to calculate the percentage of fat cell differentiation for each experimental condition. Preadipocytes: undifferentiated preadipocytes in growth media; adipocytes: differentiating preadipocytes; differentiating preadipocytes. in complete differentiation media supplemented with C5 (quercetin), C2 & C5 (luteolin & quercetin), C1, C2, C3, & C5 (berberine, luteolin, resveratrol, & quercetin), or F1 composition. The error bars indicate the standard deviations across ten field-of-views examined per experimental condition. Asterisk (*) indicates a statistical significance of p-value ≤0.01 versus adipocytes treatment condition.

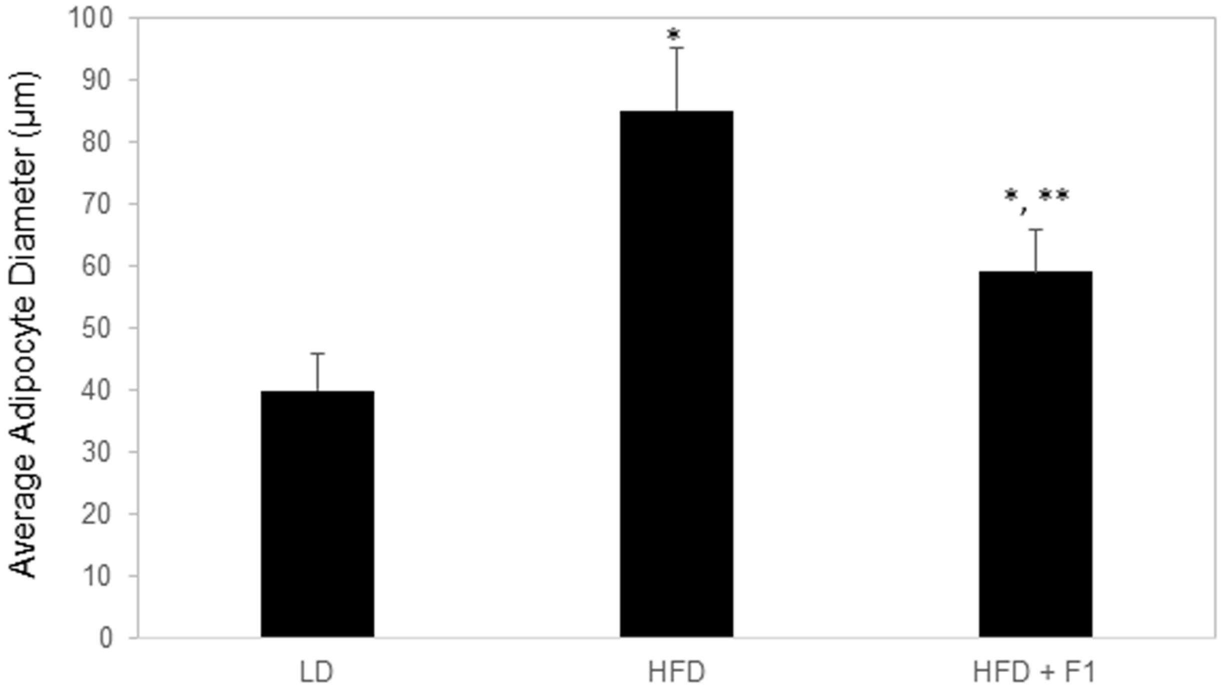

Figure S2. Average adipocyte diameter as a function of diet groups. H&E histology images of visceral adipose tissues were used for manual measurement of adipocyte diameters. The diameters of approximately 50 adipocytes were measured for each collected visceral adipose tissue, or 2000 adipocytes total for forty mice per diet group, using ImageJ software (National Institutes of Health, Bethesda, Maryland, USA). The error bars indicate the standard deviations across 2000 adipocytes measured for forty mice in each diet group. Single asterisk (*) indicates a statistical significance of p-value ≤0.01 versus the lean diet group. Double asterisks (**) indicates a statistical significance of p-value ≤0.01 versus the high-fat diet group.

Table S1. Phytonutrients for cell cultures

Phytonutrient	Cat. No.	Purity	Supplier
Berberine	10006427	≥95%	Cayman Chem, Ann Arbor, MI, USA
Luteolin	10004164	≥98%	Cayman Chem, Ann Arbor, MI, USA
Resveratrol	70675	≥98%	Cayman Chem, Ann Arbor, MI, USA
Fisetin	15246	≥90%	Cayman Chem, Ann Arbor, MI, USA
Quercetin	10005169	≥95%	Cayman Chem, Ann Arbor, MI, USA
Fucoidan	20357	≥85%	Cayman Chem, Ann Arbor, MI, USA
EGCG	70935	≥96%	Cayman Chem, Ann Arbor, MI, USA
Hesperidin	18646	≥98%	Cayman Chem, Ann Arbor, MI, USA
Curcumin	81025	≥90%	Cayman Chem, Ann Arbor, MI, USA

Table S2. Phytonutrients for dietary supplement

Phytonutrient	Cat. No.	Purity	Supplier
Berberine	BPBE-698	≥97%	Benepure, Chengdu, Sichuan, China
Luteolin	BPBE-626	≥98%	Benepure, Chengdu, Sichuan, China
Resveratrol	Resveratrol	≥98%	Nanjing NutriHerb BioTech, Nanjing, China
Fisetin	Fisetin	≥98%	Nanjing NutriHerb BioTech, Nanjing, China
Quercetin	BPBE-601	≥95%	Benepure, Chengdu, Sichuan, China
Fucoidan	Fucoidan	≥95%	Nanjing NutriHerb BioTech, Nanjing, China
EGCG	BPBE-645	≥95%	Benepure, Chengdu, Sichuan, China
Hesperidin	BPBE-607	≥90%	Benepure, Chengdu, Sichuan, China
Curcumin	BPBE-691	≥90%	Benepure, Chengdu, Sichuan, China

Table S3. Chemical structures, concentrations, and daily doses of phytonutrients

Phytonutrient	Chemical Structure	Concentration (Cell Cultures)	% of F1	Daily Dose (Mice)	Daily Dose (Human Equivalent)
Berberine	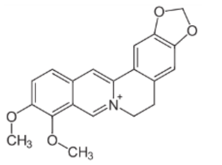	10 μM or 3.5 μg/mL	18%	36 mg/kg	2.88 mg/kg
Luteolin	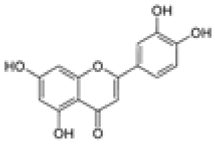	20 μM or 5.7 μg/mL	10%	20 mg/kg	1.6 mg/kg
Resveratrol	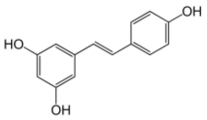	40 μM or 9.1 μg/mL	18%	36 mg/kg	2.88 mg/kg
Fisetin	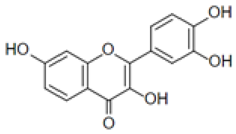	50 μM or 14.3 μg/mL	9%	18 mg/kg	1.44 mg/kg
Quercetin	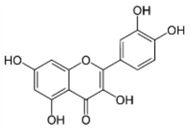	25 μM or 7.6 μg/mL	9%	18 mg/kg	1.44 mg/kg
Fucoidan	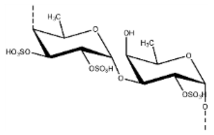	100 μg/mL	9%	18 mg/kg	1.44 mg/kg
Epigallocatechin Gallate (EGCG)	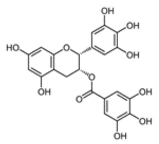	20 μM or 9.2 μg/mL	9%	18 mg/kg	1.44 mg/kg
Hesperidin	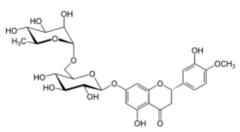	20 μM or 12.2 μg/mL	9%	18 mg/kg	1.44 mg/kg
Curcumin	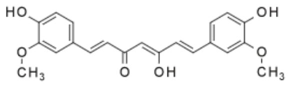	20 μM or 7.5 μg/mL	9%	18 mg/kg	1.44 mg/kg
F1	All of the above	10 μg/mL	100%	200 mg/kg	16 mg/kg
